# Fast-Growing Engineered Microbes: New Concerns for Gain-of-Function Research?

**DOI:** 10.3389/fgene.2018.00207

**Published:** 2018-06-29

**Authors:** Lei Pei, Markus Schmidt

**Affiliations:** Biofaction KG, Vienna, Austria

**Keywords:** fast-growing, synthetic biology, biosecurity, vaccines, *Mycoplasma pneumoniae*

## Abstract

Research on fast-growing microbes holds promise for many industrial applications, including shortening test and trial times in research and development stages and reducing the operation costs for production. Such microbes can be obtained either by selecting naturally occurring variants or via metabolic engineering approaches, either eliminating ‘unnecessary’ or adding necessary pathways affecting growth speed in the cell. Here, we review recent research and development of engineered fast-growing strains in industrial biotechology, with a special focus on vaccine production using (synthetic biology) engineered pathogenic strains. We will discuss whether this represents a security concern and whether the industrial biotech sector needs to pay more attention to issues of Gain-of-Function (GoF) while developing and harnessing these fast-growing microbes. We will also shed a light on the use of in-built biosafety circuits as a way to control the propagation of fast-growing strains, including their capacity to survive in the environment. Other possible GoF concerns raised by the publication of research results in this field will be also addressed. In conclusion, judging from the current development from the field, assessing the potential GoF risks on engineered fast-growing microbes does not lead to a clear generalized outcome. We argue that fast growing strains need to be evaluated in combination with their wild type and engineered characteristics, and require always a case-by-case assessment. Monitoring the progress of the field and proactively raising awareness on the GoF issues among the scientists are important for the further development of the field.

## Introduction

Prosperity and material wealth in modern society is based on economic growth, innovation and improving the efficiency of production processes. The Knowledge-Based Bioeconomy (KBBE), aims to harness the innovations from biotechnology to produce sustainable products and processes, is no exception with its continued success depending on the creation of new and useful products and processes that are further improved on a continuous basis, ranging from producing sustainable biofuels to biological processes for bulk chemical production to fine chemicals. Synthetic biology is considered a key engineering discipline driving the bioeconomy forward, and one of its goals is to obtain organisms with a high replication rate, by either searching for novel natural species with fast generation times, further engineering those ones which are known for fast growth, or engineering those species known for slow growth. Using engineering approaches to enhance growth rate in a target organism, however, is considered as a Gain-of-Function (GoF) of an organism, which means that the engineered microbe has qualitatively and quantitatively different traits than the wild type that is usually used to describe the biosecurity (and biosafety) risks. GoF, therefore, needs to be examined in regarding novel risks for its intended and unintended applications. In this mini-review we will look at a number of selected GoF examples and reflect on their risks.

Cellular growth rate is determined by a variety of factors, spanning both physiological processes (intrinsic) and growth conditions (extrinsic). Recent developments in biotechnology have provided approaches to enhance growth rate by optimizing growth conditions, leaving room only for further improving growth by engineering physiological processes. Although random mutagenesis has been applied to generate mutant strains that can grow fast, the development of novel technologies (e.g., metabolic engineering, bioinformatics, systems and synthetic biology, and genomics) make it possible to engineer the physiological processes in a more rational way.

Generally speaking, a strain known for fast growth in one species may already have optimized its physiological processes for fast growth through evolution, but some processes may still be open for further improvement. Other than improving the growth rate further, utilizing rapid growth processes for useful applications is also important – such as novel chassis organisms for industrial product processes, or improved vaccines. We will review the recent achievements of research development for microbes in fast-growing variants.

To gain a better underpinning knowledge of fast-growing microbes, genome-wide studies were conducted to predict the highly expressed genes from full genomes of four fast-growing bacteria: *Escherichia coli, Haemophilus influenzae, Vibrio cholerae*, and *Bacillus subtilis* (Karlin et al., 2001). The predicted highly expressed genes (PHX) were identified as those for ribosomal proteins, major transcription/translation processing factors, major chaperone/degradation proteins, essential enzymes for energy metabolism pathways, as well as enzymes for biosynthesis of fatty acids, amino acids, and nucleotides. Approximately, 60 genes were common to the four fast-growing bacteria belonging to the PHX family. The findings from these bacteria indicate a promising approach in reconstructing the genome of other microbes.

## Fast-Growing Strains for Research Purposes and Industrial Applications

Fast growth and, hence, a reduced replication rate is one of the aims of modern biotechnology. The reasons why fast growth is desired differ depending on the overall goal of the bioengineers. The following non-exhaustive description of currently ongoing R&D dealing with fast-growing strains is meant to give an overview about the diversity of species and approaches used to come up with faster growth. While yeasts are mainly engineered to enhance production of certain biomolecules, prokaryotic pathogens are modified to better understand pathogenicity or to develop effective vaccines.

### Kluyveromyces marxianus

Yeasts are popular industrial production strains with some preferable features over prokaryotic hosts, for example, they are better to produce human proteins due to their post-translational modifications similar to the human’s. Among them, *Kluyveromyces marxianus* is one of the well-known fast-growing yeasts. A method termed ‘cell selection’ to identify cellular sites controlling cell growth has been developed to select for highest growth rate on defined mineral medium, although no direct modification on the microbial genome was conducted (Groeneveld et al., 2009). A yeast strain with 30% increased maximum growth rate (shortening from 0.6 to 0.8 replications per hour, or approximately 52 min) was identified by applying pH-auxostat cultivation (constant pH at 4.5) to a culture for a long time (in 50-generation time). It showed that an increase of growth rate could be achieved by increasing membrane surface area (a 0.7% increase in growth rate and a 1% increase in membrane surface area). Using systemic biological approaches to design metabolic engineering pathways to simultaneously activate membrane processes may help to increase further growth of the eukaryotic cells.

### Saccharomyces cerevisiae

*Saccharomyces cerevisiae* is one of the most common strains used for industrial production. A comprehensive understanding on how growth rate is controlled is important to the development of a fast-growing variant of this species. It is known that growth rate is regulated by a set of genes to adapt to the environmental conditions. Studies on different auxotrophic yeast strains revealed that essential genes related to cell growth might be regulated by more complex cellular processes as well as environmental conditions (Mulleder et al., 2012). A set of genes was identified in the genome of *S. cerevisiae* that controls the growth rate in response to growth conditions (Pir et al., 2012). Studies on the mutants of growth-regulated genes showed that the mutants grown at the maximum growth rate resulted from “a trade-off between the selective advantages of rapid growth and the need to maintain the integrity of the genome” (Pir et al., 2012). To study the genetic interactions on a genome-wide scale, approaches to map the networks of *S. cerevisiae* have been developed (Lin et al., 2008; Vizeacoumar et al., 2010). A comprehensive understanding on how complex genetic interactions affect growth phenotype would help to rationally design yeast strains with optimized production yields for industrial applications, particularly those ones with increased maximum growth rate to produce biomass in a rapid speed. Studies have been conducted to reveal the genetic interactions and their impact on growth phenotypes, as well as to improve the ability to *in silico* predict the growth phenotype of *S. cerevisiae* (Dikicioglu et al., 2013). Other than to make yeast grow faster, the research on the growth of brewer’s yeast is also aimed at harnessing the knowledge of essential genes for growth control to use as biocontainment strategy for genetically engineered yeast, targeting yeast transcriptional profile and the expression of site-specific recombinase. Two types of safeguard switches (triplex histone switch and recombination-induced lethal switch) were implemented in *S. cerevisiae*, controlling one or more essential genes with escape frequencies of less than 10^-6^ (Cai et al., 2015).

### Vibrio natriegens

*Escherichia coli* is one of the model (host) organisms for recombinant gene technology. Yet its growth rate, approximately 20 min per generation in standard growth condition (Sezonov et al., 2007), is becoming less ideal for the increasing demand of rapid turnover times in some applications. Thus, researchers have turned their interest to find new chassis organisms with more rapid growth rates. *Vibrio natriegens* is one of the novel chassis organisms of interest. This Gram-negative bacterium is known for its fast generation time, half of that of *E. coli*, of less than 10 min (Eagon, 1962). A complete *V. natriegens* genome is now available, consisting of two chromosomes of approximately 3.2 and 1.9 Mbp respectively, encoding more than 4500 open reading frames (ORFs) (Maida et al., 2013). *V. natriegens* compatible genetic tools and techniques are also developed to allow using this organism as an engineering host (Lee et al., 2016). The genetic tools and techniques are a DNA transformation protocol, plasmids and bacteriophages replicated in *V. natriegens*, a whole-genome transposon mutagenesis (WGTM), and a CRISPR interference system in *V. natriegens* (Lee et al., 2016). In particular, a transposon library was generated by WGTM to be screened for mutant strains that could grow even faster, although no mutant could be identified from this library (Lee et al., 2016). As the bacteria with the shortest replication time known, its ability to survive on low cost carbon sources and its ability to secrete proteins into the medium makes *V. natriegens* a promising candidate as a biotechnology chassis (Weinstock et al., 2016).

### Mycobacterium tuberculosis

*Mycobacterium tuberculosis* is a pathogen causing tuberculosis (TB). Due to increasing cases of multi-drug resistant strains reported worldwide, novel antibiotics and vaccines are urgently needed to combat the infections (World Health Organization [WHO], 2016). Although mycobacterial genetics have been unveiled for years, genetic manipulation of mycobacterial genomes, particularly of *M. tuberculosis*, remains difficult partly due to the slow growth rate and lack of proper genetic modification tools. One bottleneck issue is the slow growth of the organism, a doubling time of which could reach up to 69 h. Using a fast-growing variant might help the researchers shorten their research cycle. Yet, the growth rate issue is more complicated. It is known that *M. tuberculosis* is a persistent pathogen due to its unique cell wall composition and asymmetric growth in the host, which might be a plausible reason for clinical latency (Kieser and Rubin, 2014). The heterogeneity of growth might lead to the development of antibiotic tolerance and persistence of the pathogen (Aldridge et al., 2012; Joyce et al., 2012). Lack of proper genetic tools is another issue hindering the research. A reliable modification of *M. tuberculosis* genes has been critical to study the functions important for the growth and pathogenicity of the organisms. The *M. tuberculosis* compatible genetic delivery systems are non-replicating vectors and mycobacterial phages (e.g., Che9c phage RecET recombination systems) (Murphy et al., 2015) for the introduction of target gene elements into *M. tuberculosis*, harnessing the endogenous recombination systems to integrate the targets into the chromosome in order to achieve the desired genome modifications – deletion, site mutation, or insertion. System biology approaches have been applied to compare the genomes of the *M. tuberculosis* (approximately 4 Mbp, doubling time around 20–24 h in culture condition), *M. leprae* (approximately 3.2 Mbp, an even slower growth, with a doubling time of around 14 days), and the fast-growing non-pathogenic species *M. smegmatis* (approximately 7 Mbp, doubling time around 3–4 h in culture condition) (Akinola, 2013). Although the growth rates of these three species are quite different, approximately 1000 proteins are shared by them (Akinola, 2013). Studies have been conducted to investigate the genetic requirements for fast (doubling time of 23 h) and slow growth (doubling time of 69 h) of mycobacterium in a carbon-limited chemostat condition. A transposon mutant library was generated by using chemostat culture to screen for the genes controlling the slow and fast growth in *Mycobacterium bovis* (strain BCG). The results showed that 84 genes were specific for slow growth and 256 genes were required to switch to fast growth, suggesting that growth rate control in mycobacterium was fine-tuned by a large set of genes ranging from genes encoded some virulence determinants, regulation elements, to metabolic enzymes (Beste et al., 2009). Further analysis of these genomes might provide clues to identify genes that promote growth rate in the mycobacterium species and to develop fast-growing variants of *M. tuberculosis* for research purposes.

### Mycoplasma pneumoniae

*Mycoplasma pneumoniae* contains a very small genome, with only 816,394 base pairs (Himmelreich et al., 1996). Due to the small genome size, the genus of mycoplasma has been studied extensively, particularly the genomes of *M. genitalium, M. mycoides*, and *M. pneumoniae*. The bacteria from this genus attracted attention, not only as model organisms for the chemical synthesis of a full genome (Gibson et al., 2008; Lartigue et al., 2009; Hutchison et al., 2016), but also as model organisms to study factors relevant to cell growth. Among them, *M. pneunomiae* is of particular interest for cell growth. Despite its very small genome, *M. pneumoniae* is not simple – the cellular functions are rather complex, e.g., many *M. pneumoniae* proteins are part of more than one cellular machine or protein complex (Juhas et al., 2011). It is predicted that the genome of *M. pneunomiae* contains 677 ORFs, more than 75% of which show similarity to genes/proteins of other organisms. The alignment of the genome of *M. pneumoniae* with other organisms revealed that the reduced genome size of *M. pneumoniae* might result from the evolution of ancestral bacteria losing certain anabolic and metabolic pathways. Thus, although it can self-replicate, *M. pneumoniae* requires exogenous essential metabolites to survive (e.g., a certain amino acids and lipids). Investigating the co-evolution of mycoplasma and its hosts that led to a reduction of genome size, might hold the key to understand its relative slow growth. Artificially reduced genomes, in contrast, have recently been shown to exhibit a faster growth and higher mutation rate, demonstrating that there is no simple relation between genome size and growth rate (Nishimura et al., 2017). To better understand the minimal cellular machinery required for life, tandem affinity purification-mass spectrometry (TAP-MS) was applied to study the proteome organization in a genome-reduced bacteria based on the genome of strain M 129 (Kuhner et al., 2009). 62 homo-multimeric and 116 hetero-multimeric soluble protein complexes were identified, of which more than half were novel. By combining the pattern recognition and classification algorithms, the protein complexes identified by TAP-MS could be underpinned within the whole cell, while matching with the existing electron tomography. This knowledge from proteomics would help to better pinpoint the genes for cellular function, which could be harnessed for complementing the lost or weakened cellular functions linked to slow growth. The cellular blueprint of *M. pneumoniae* could allow scientists to complement those elements needed to facilitate cell growth by full analysis *in silico* of essentiality, as well as metabolomics, transcriptomics, and proteomics data (Eisenstein, 2017). All findings on the essential genes and interactions on metabolic and transcriptional networks provide clues of counter strategies to compensate the lost cellular function resulting from evolution – adding supplement genes or elements to the already reduced genome in order to enhance the growth rate. These strategies include supplementing the organisms with growth related genes from the fast-growing strain from the genus of mycoplasma or other fast-growing microbes (e.g., the common genes PHX, found in other fast-growing bacteria, as mentioned above), and complementing those metabolic functions the slow growth strains lack, such as lipid synthesis.

**FIGURE 1:**
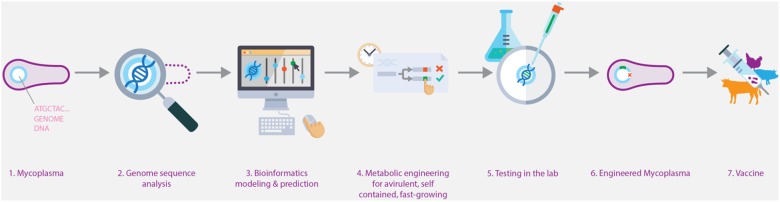
Overview on using synthetic biological approaches to engineer mycoplasma vaccine strains with fast-growing properties. Image created by Biofaction, and used with permission.

The European Commission funded Horizon 2020 project “MycoSynVac”^1^ is developing animal vaccines based on highly engineered pathogenic mycoplasma strains. To reach this goal, a number of obstacles have to be overcome, such as reducing pathogenicity, developing fast-growing strains for efficient production, implementing biosafety circuits to better control the new strains and developing a set of genetic engineering techniques (**Figure 1**).

In order to make a safe vaccine chassis, the possible virulence factors from the wild type strain were identified by sequencing the genomes of 23 clinical isolates of *M. pneumoniae*. Comparing the single nucleotide polymorphisms (SNPs), non-synonymous mutations, indels, and genome rearrangements of these clinical isolates and the other four standard reference sequences, new subclasses were identified within the two main clinical classes (types 1 and 2), while several putative virulence factors and antigenic proteins were found from the sequences of these clinical strains (Lluch-Senar et al., 2015).

To tackle the issue that *M. pneumoniae* is difficult to genetically modify, new genetic tools are in development. In-yeast engineering of bacterial genome is one of those, aiming to make editing mycoplasma genome possible with the newly developed CRISPR-Cas9 approach. As a proof of principle, the mycoplasma glycerol-3-phosphate oxidase-encoding gene (*glpO*) was deleted in yeast by the action of Cas9 guided by a 90-nucleotide-long RNA; and the edited genome was transplanted back into a recipient mycoplasma cell subsequently, paving the way for further genome editing in mycoplasma to develop fast-growing variants using whole genome synthesis or editing approaches (Tsarmpopoulos et al., 2016).

Other than searching for genes to enhance cell growth, studies are also conducted to identify genes essential to a minimal genome by comparing genomes of mycoplasma with the latest version of the smallest self-replicated genome JVCI-Syn3.0 (Kamminga et al., 2017). This could convert into knowledge leading to *in silico* design for fast-growing mycoplasma. In addition, multiple layers of biosafety strategies are tested in *M. pneumoniae* as well, implementing the biocontainment strategies developed for other engineered microbes (Wright et al., 2013, 2015), which would help to make the novel mycoplasma vaccine chassis not only easier and faster to produce but also safer.

## GoF Issues Raised From Microbes With Fast-Growing Properties

There is no exclusive heuristic to identify whether or not a modification in a biological system causes biosecurity concerns, but a number of recommendations, guidelines and regulations exist to help sort out that question.

Among them, the so-called NAS Fink Committee suggested seven types of experiments of concern that would give reason to precaution (NAS, 2004). According to them, experiments of concern are those that:

(1) “Would demonstrate how to render a vaccine ineffective;
(2) Would confer resistance to therapeutically useful antibiotics or antiviral agents;
(3) Would enhance the virulence of a pathogen or render a non-pathogen virulent;
(4) Would increase transmissibility of a pathogen;
(5) Would alter the host range of a pathogen;
(6) Would enable the evasion of diagnostic/detection tools;
(7) Would enable the weaponisation of a biological agent or toxin.”

In addition to these experiments of concern that focus on the risks that biotechnology and life science would unknowingly and without intention create tools for biowarfare, another concern lies in the easiness and efficiency of producing a large number of bioweapons.

One concern with GoF is the enhanced up-scaling of productivity granted by using fast-growing microbes (Giersch and Schmidt, 2010). We describe the development of fast-growing *S. cerevisiae* variants as an example to explain the possible GoF concerns from this type of research. *S. cerevisiae* is a ‘generally recognized as safe’ (GRAS) organism. A genetically modified *S. cerevisiae* with fast-growing property would be of great interest to industry using the yeast as a starter, because it would make the fermentation process more cost efficient than those processes with (only) optimal physical growth conditions. Yet, the technologies or principles applied to develop such fast-growing strains would also be feasible/useful to develop yeast strains that produce biomolecules of concern. Engineered yeast strains to produce illicit compounds are already developed, although the yield is still low (Galanie et al., 2015). A fast-growing *S. cerevisiae* strain could be further modified to produce opioids or toxic biologicals alike. It would a raise burden for the control of prohibited biologicals. Thus, the most likely GoF challenge of developing fast-growing microbes would be, if individuals with ill intentions gain access to these fast-growing microbes and turn them into a workhorse to produce biological toxins that could be used as bioweapon. In this possible scenario, the fast-growing microbes alone would not be sufficient to produce any dangerous substance, but would allow a more efficient production process.

A second GoF issue raised by engineered fast-growing microbes would be some of the genetic elements tested to enhance the growth rate, taking for example the engineering of *V. natriegens*. To pursue strains that could grow ever faster, the replicon of vibrio-phage CTX, often studied in the context of pathogenic *V. cholerae* was tested and found compatible with *V. natriegens*, although the infection rate of the phage itself to the target host was 100 fold lower than the native host *V. cholerae* (Lee et al., 2016). The safety issue was discussed in the same study, arguing that the free form of CTX virions was low in environment and no replicated virion was produced by lab-based transformation (by direct electroporation). Still, it could not rule out the possibility of the fast-growing strains gaining toxicity (due to the known compatibility of the replicon of the vibriophage) while they would have been released to the environment. For a bioterrorist, such knowledge could be further applied or developed to generate fast-growing microbes causing cholera-like syndromes, which might offer them new paths to generate novel pathogens for epidemic or even pandemic infection. Overall the risks seem rather low for this scenario as well, since a number of additional modifications, unrelated to fast growth, would be needed in *V. natrigiens* to become problematic.

The third GoF issue of fast-growing microbes concerns the research on making (originally) pathogenic microbes grow faster for research and/or vaccine production purposes, as in the case of mycoplasma. Given established evolutionary principles, there must be a balanced interaction between the infected host and the pathogenic microbes with slow growth – the microbes grow at a slow rate in order to not kill their hosts rapidly, to be persistent and/or to co-exist with the hosts. Within the genus of mycoplasma, there are fast-growing species that are mostly less virulent than the slow growth species. And the cell growth related genetic elements from these fast-growing species would be the elements that would graft to the slow growth species, taking examples of the research on fast-growing mycobacterium and mycoplasma strains. The risk from this type of research is that, if such balance is altered by granting the pathogenic organisms a fast-growing feature, the pathogenesis of infection would also change, with unknown consequences. Thus, the engineered fast-growing variants of the pathogenic species would have to be assessed on how virulent the fast-growing variants are compared to their wild type species in the host or potential host. The assessments of the potential newly gained pathogenesis should include both *in vitro* and *in vivo* analysis, as well as environmental assessments (the ability for pathogens to survive outside human hosts). Given how complicated and expensive it is to conduct these assessments, it would be better to take the security issues into consideration beforehand and implement the built-in safety controls. Some of the fast-growing microbes in development were also tested with built-in genetic safety guards as mentioned above for *S. cerevisiae* and *M. pneumoniae*. The GoF concern on this type of research might come from the possible unintended outcome of novel vaccine development- when turning (relatively) slow growing pathogens into fast-growing non-pathogenic variants, one can not rule out the risk that somewhere in the development process (or afterwards) a fast-growing pathogen could be created unintentionally. Given that very few pathogens get their pathogenicity via fast growth, and that many pathogens use slow growth as their evolutionary strategy, we do not expect this to be a major issue. To be sure, however, the R&D process should be aware of the possibility of this risk and implement measures to detect and minimize these risk.

## Conclusion

Assessing the potential GoF risks on engineered fast-growing microbes does not lead to a clear outcome that can be applied to all types of engineered fast-growing strains. The approaches used in synthetic biology to generate the fast-growing strains are quite different from traditional approaches (e.g., screen or select natural variants, optimal growth conditions, etc.) and research has not yet produced clear evidence for a deterministic effect on growth. These novel approaches are so specialized and with very little accumulated knowledge that it is not easy to assess the potential risks. Also, the reasons and types of modifications differ substantially, ranging from robust production of biomolecules for industrial applications to the generation of new vaccines. A closer look at some of the products involving fast growth could pose GoF concerns, in case fast growth would be combined with other experiments of concern or for nefarious purposes. Research on fast growth by itself does not seem to exhibit significant high risks *per se*. GoF risks, however, are not totally negligible either, as fast growth could be one of many pieces of a puzzle to produce dangerous pathogens for humans, livestock or crops in an efficient and fast way. Therefore, in the absence of a clearly visible single high risk from a fast-growing chassis, but given their potential contribution for biotechnology production systems, the only meaningful recommendation to give at this point is to keep assessing the GoF of fast-growing strains on a case-by-case basis and maintain the constant monitoring of developments in the field while proactively raise awareness among scientists (NAS, 2004).

## Author Contributions

LP carried out research on fast growing strains in the species mentioned and drafted the manuscript. MS had the initial idea of identifying biosecurity/biosafety related risks of fast growing strains in connection to gain of function, he was also mainly responsible for discussion and the biosecurity interpretation of information gathered about fast growing strains.

## Conflict of Interest Statement

The authors declare that the research was conducted in the absence of any commercial or financial relationships that could be construed as a potential conflict of interest.

## Footnotes

1 www.mycosynvac.eu
